# Chirality effects on an electron transport in single-walled carbon nanotube

**DOI:** 10.1038/s41598-020-76047-9

**Published:** 2020-11-03

**Authors:** J. Charoenpakdee, Ongart Suntijitrungruang, S. Boonchui

**Affiliations:** 1 Department of Physics, Faculty of Science, Kasetsart University, Bangkok, 10900 Thailand; 2 Center of Rubber and Polymer Materials in Agriculture and Industry (RPM), Faculty of Science, Kasetsart University, Bangkok, 10900 Thailand

**Keywords:** Materials science, Physics

## Abstract

In our work, we investigate characteristics of conductivity for single-walled carbon nanotubes caused by spin–orbit interaction. In the case study of chirality indexes, we especially research on the three types of single-walled carbon nanotubes which are the zigzag, the chiral, and the armchair. The mathematical analysis employed for our works is the Green-Kubo Method. For the theoretical results of our work, we discover that the chirality of single-walled carbon nanotubes impacts the interaction leading to the spin polarization of conductivity. We acknowledge such asymmetry characteristics by calculating the longitudinal current–current correlation function difference between a positive and negative wave vector in which there is the typical chiral-dependent. We also find out that the temperature and the frequency of electrons affect the function producing the different characteristics of the conductivity. From particular simulations, we obtain that the correlation decrease when the temperature increase for a low frequency of electrons. For high frequency, the correlation is nonmonotonic temperature dependence. The results of the phenomena investigated from our study express different degrees of spin polarization in each chiral of single-walled carbon nanotube and significant effects on temperature-dependent charge transport according to carrier backscattering. By chiral-induced spin selectivity that produces different spin polarization, our work could be applied for intriguing optimization charge transport.

## Introduction

Carbon nanotube (CNT) photonics is an emerging field with fascinating properties^1–4^. It offers a challenge to both fundamental and applied sciences. Many properties of CNT are related to its lattice structure, that allow for modulating charge dynamics through the curvature of the nanotube surface. Physical framework in condensed matter nature is discussed such as narrow-linewidth blackbody emission^5^, the first electrically driven ultrafast CNT light emitter^6^, electronic many-body correlation effects^7^, etc. With interesting properties, the heat equation and Planck’s law was used to study the nanotube lamp fabrication process^8–11^. In integrated micro-sized light sources for photonic and optoelectronic integrated circuits, and optical interconnection is applied by exploiting CNT devices with narrow line width. A one of the important properties in the twisted carbon is chirality. With the effect of chirality, many microscopic phenomena take place. For instance, the graphene nanoribbons with various edge orientations (chirality) exhibit edge-dependent electronic and optical selection rules^12–15^. The efficiency of absorption and emission of different chiralities CNT was considered^16,17^. The role played by the type of surfactant, surfactant concentration, and nanotube concentration on the efficiency of absorption and emission is discussed. Other exciting properties occur when the curvature is presented. For an electrical conductivity of single-walled carbon nanotube (SWCNT), its anisotropy lattice structure effects in the electron transport of SWCNT are considered in many research works. For example, electron transport along a chiral trajectory is decomposed into current components along the tubule axis and its circumference^18,19^. By the Boltzmann kinetic equation, Slepyan had shown that the impedance for different SWCNT’s (zigzag, armchair, and chiral) is derived as a result of the dynamic conductivity^20^. For metallic chiral, the longitudinal conductivity is proportional to the inverse of the radius tube. Nonlinear electron transport and chiral effects on the magnitude and the direction of the total time-averaged current were predicted^21^. The theory of photogalvanic effects was considered for particular chirality effects^22^.

Spin–orbit interaction (SOI) plays an essential role in the electronic and optical properties of solid-state systems. For examples, group-IV monoelemental 2D honeycomb materials beyond graphene, such as silicene, germanene, and stanene, have been predicted to exhibit band gaps, depending on the strength of SOI^23^. The binary compounds of group III–V elements have also been proposed as the honeycomb lattices with large energy gaps^24–26^. For theoretical discussion, the SOI is regarded in many properties of SWCNT in^27^. The velocities of the Dirac particle is asymmetric due to these effects, showed by Izumida et al.^28,29^. Electron transport in the potential step, which mimics a long-range potential in the armchair nanotubes with curvature induced SOI, is investigated^30^. Other property of SWCNT caused by the intrinsic magneto-optical properties of suspended SWCNT were studied for various chiralities essential on the signature of spin orbit coupling^31^. A full analysis of spin orbit interaction in SWCNTs requires, however, to examine the impact on electron transport in the antisymmetric group velocity of honeycomb carbon lattice of a tube. Carbon 2p orbitals presenting a weak spin–orbit interaction in SWCNT was discussed^32–34^. Since the effect of this interaction is small, It needs to consider its effects on the lowest order of energy dissipation. In particular, carbon nanotubes applied by the magnetic field make breaking all of these symmetries, and the spin–orbit coupling is considerable. The SOI is directly observed as a splitting of the four fold degeneracy of a single electron in ultra-clean quantum dots^35^. Although the atomic spin–orbit coupling in carbon is weak, the spin orbit coupling in carbon nanotubes can be significant due to their curved surface. However, the spin–orbit coupling in the carbon nanotube devices that is an order of magnitude larger than previously measured was reported^36^. Experimentally^37,38^, in a recent experiment, DNA-carbon nanotube spin filters in which carbon nanotube have been functionalized with two different classes of sequences, exhibiting different degrees of interaction with the carbon nanotube is reported^39^. This work shows that chirality-induced spin selectivity induce different degrees of spin polarization in the channel, with a significant impact on temperature-dependent charge transport and interference phenomena arising from carrier backscattering. To understand chirality-induced spin selectivity or spin filters effect, it implies an intriguing ways to control carrier transport at the nanoscale and the realm of mainstream spintronic devices.

In this paper, we theoretically studied the characteristics of conductivity for SWCNT caused by SOI. This paper is organized as follows: we begin with results, and discussions of characteristics of the retarded current–current correlation function depending on the chiral. We show the results of calculations. It implies that the significance of spin polarization depends on each chiral of SWCNT and temperature-dependent charge transport. We, furthermore, interpret our simulation results in physical means that thermal energy boost the electron’s energy. In certain cases at specific frequencies, the characteristic of temperature dependence are nonmonotonic as a result of conduction energy band’s attribute. In “Conclusions”, our conclusion is shown. Finally, a theoretical method of the tight-binding model and current operator in a chiral SWCNT are shown. We demonstrate that characteristics of the retarded current–current correlation function depend on the chiral indexes.

## Results and discussions

**Figure 1:**
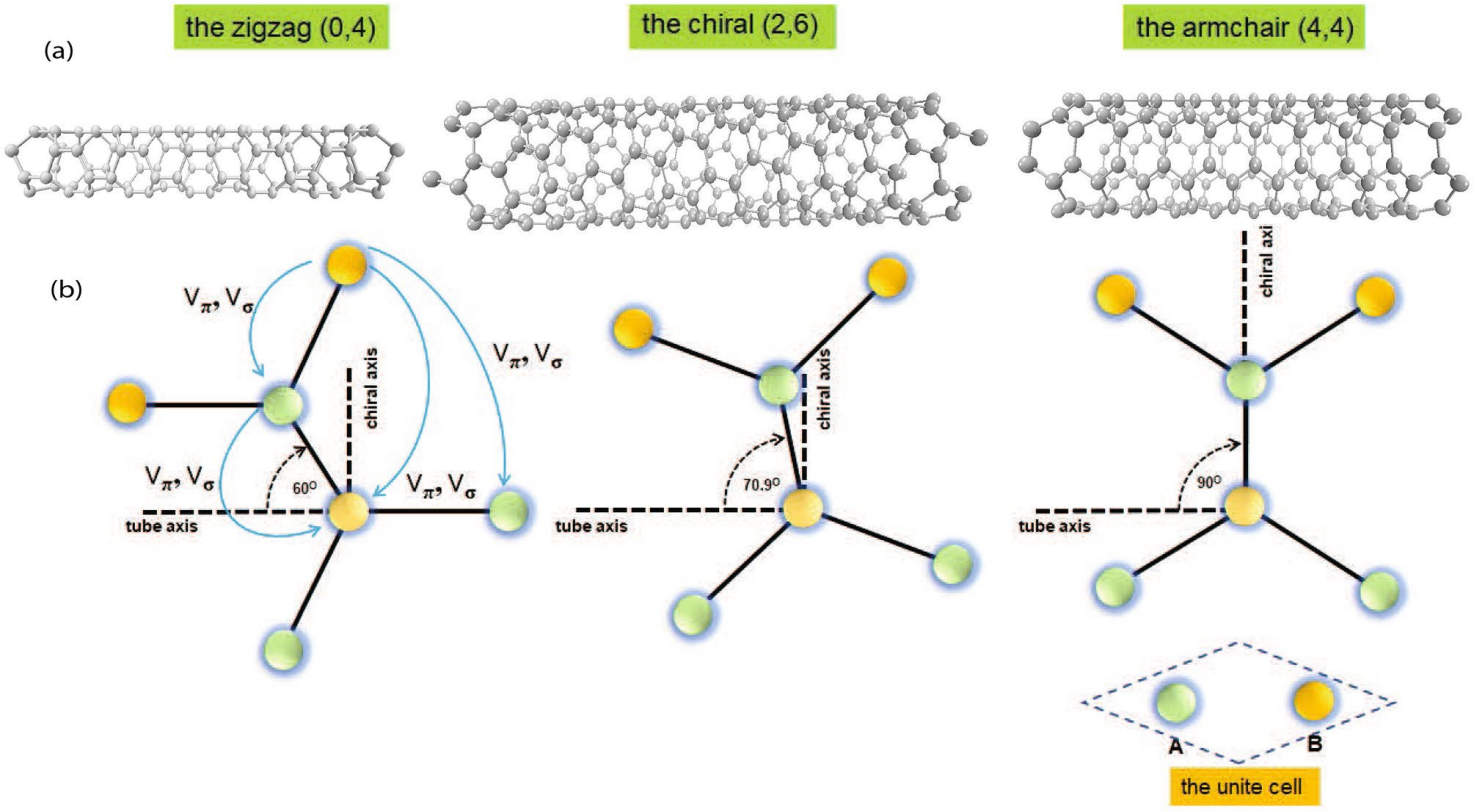
(**a**) Molecular structures of three SWCNT forms that are the zigzag (0,4), the chiral (2,6), and the armchair (4,4). (**b**) This figure shows that its molecular bond angle of each form. These are the components of the nearest-neighbour lattice vector $\overrightarrow {\delta }$ in the basis of direction perpendicular and parallel to the tube axis.

In order to theoretical explore properties in SWCNT, we consider SWCNT with the diameter and the chiral angle as the zigzag SWCNT, (0,4) $( \rho =0.313 \, {\text {nm}}, \theta =0^\circ )$, the chiral SWCNT (2,6) $(\rho =0.565 \, {\text {nm}}, \theta \approx 19.1^\circ )$, and the armchair SWCNT, (4,4) $( \rho =0.542 \, {\text {nm}}, \theta =30^\circ )$ as shown in Fig. 1a. Our calculations begin from considering the tight-binding model that the hopping integral between the $\pi$ orbital is modified and added the curvature-induced $\sigma -\pi$ due to tilted Dirac cones near the *K* point. We examine the results of SOI for each chiral affecting the motion of electron spin up, which is along $+z$ tube axis. We have shown that specific curvature induces the asymmetry conductivity of three types of SWCNT for positive and negative wave vectors, corresponding a shift of the Dirac point. By shifted the Dirac point that makes asymmetric heat transfer, asymmetric electrical conductivity occurs. According to experiment work^40^, in the certain chiral indexes of SWCNT, the asymmetric conductivity exists. Such phenomena is consistent with particular experiments on heat transfer. In 2006, A relevant attempt to build a thermal rectifier was based on a graded structure made of carbon and boron nitride nanotubes that transport heat between a pair of heating/sensing circuits. The resulting nanoscale system yields asymmetric axial thermal conductance with higher heat flow in the direction of decreasing mass densitys^41^. On the other hand, the thermal rectification phenomenon in a single-wall mass graded carbon nanotube was investigated by molecular dynamics simulation. The dependence of the rectification factor R on temperature, nanotube diameter, and length, as well as the mass gradient is obtained^42^. The dependence of the rectification factor R on temperature, nanotube diameter, and length as well as the mass gradient is obtained.

Moreover, our simulations demonstrate that effects from the spin–orbit coupling depend on the distance between the interacting atoms projected in the axial direction for each chiral SWCNT as a schematic diagram in Fig. 1b. The transfer integral treated follow the approach of Ando^43^ is modified $\pi$ state with $\sigma$ state. The hopping integral the neighbor orbitals induced by SOI can be written in terms of the distance $d^{||}_{\delta }$ as in Eq. (6). The strength of SOI is proportional to the distance $d^{||}_{\delta }$. When the distance close to zero, the separation of the conductivity vanish as it is shown in the case of the armchair SWCNT. Typical temperature-dependent spin polarization is shown according to the results of current–voltage (I–V) characteristics in carbon nanotube measured by Rahman^39^. For this recent experiment, they show that the SOI induce different degrees of spin polarization in the channel, with a significant impact on temperature-dependent charge transport and interference phenomena arising from carrier backscattering. Chirality-induced spin selectivity induced spin polarization is present. The experiments show the emergence of strong chirality-induced spin selectivity at lower temperatures. We theoretically studied characteristics of the spin polarization, which is estimated from the difference of the retarded current–current correlation function for the orbital $\ell =1$ and $\ell =-1$, consisting of the experiment.

**Figure 2:**
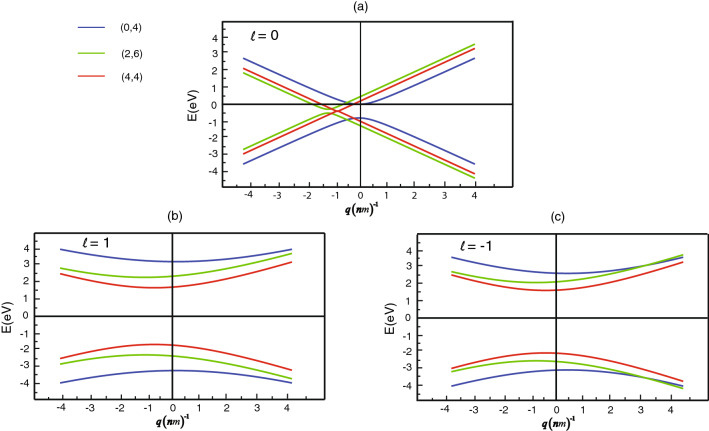
Shifted K Point of the Dirac cones for the Zigzag, the chiral and the armchair in each orbinal number (**a**) $\ell =0$, (**b**) $\ell =1$ and (**c**) $\ell =-1$.

In our numerical calculations, we choose all other parameters given as calculation following Ref.^21^. Let us start to consider plot energy band structures for a range of nanotube diameter and a series of chiral angle. Rolling graphene to a tube imposes additional periodic boundary conditions on the wave functions, leading to the quantization of momentum in the transverse component of the vector $\vec{q},q_{ \bot }  = 2\pi \ell {\mid }C_{{nm}} {\mid }$, with $\ell$ being an integer. If quantization lines run straight through Dirac points, then the line is a linear dispersion relation and zero band gap is obtained. However, if the lines bypass the Dirac points with separation, then a pair of hyperbolas with a bandgap is obtained. We attend the dispersion in the region $\approx (-4,4)$ eV. Schematics of the quantization lines [blue (0,4), green (2,6), red (4,4)] are showed in the left of the figure. First, we consider a dispersion relation for an electron with a spin-up state, which is oriented parallel to the z-axis (tube axis). In the case of the quantization lines $\ell =0$, the armchair (4,4) is gapless. But the energy gap occurs for the zigzag (0,4) and the chiral (2,6) as is shown in Fig. 2a. A minimum point of the band slides to the left hand side in case of the armchair (4,4) and the chiral (2,6). For the quantization lines $\ell =\pm 1$, the energy gap exists for all each SWCNT shown in Fig. 2b,c. To discuss the left-going (right-going) particle velocities, it is obvious from Fig. 2b,c that linear band tilting, that is, a different slope for linear bands, is seen especially for the armchair SWCNT but not for the zigzag SWCNT and the chiral SWCNT. The group velocity has the asymptotic velocity of the conduction and valence bands resulting in a linear dispersion. Here we define the velocities from Fig. 2, which are given by Eq. (20). we, now, can estimate the ratio of velocities to be $|v_{R}^{K}/v_{L}^{K}|$. In Fig. 2b,c, It signify that the maximum ratio of velocities is about 0.99, 1.98, and 3.73 for the zigzag (0,4), the chiral (2,6), and the armchair (4,4), respectively. In consequence of the different velocities, the correlations $\pi ^{(R)}$ and $\pi ^{(L)}$ are not symmetric around the minimum point of the band, thereby causing unique characteristic of the conductivity.

**Figure 3:**
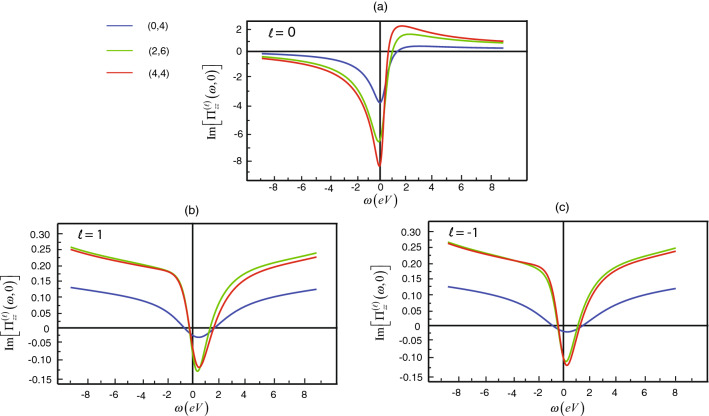
The simulation plots for the imaginary part of the current–current correlations $\Pi ^{(\ell )}_{zz}(\omega ,0;T)$ relating to the frequency $\omega$ in the zigzag (0,4) (blue lines), the chiral (2,6) (green lines), and the armchair (4,4) (red lines) with different orbital number where (**a**) $\ell =0$, (**b**) for $\ell =1$ and (**c**) for $\ell =-1$.

Next, we will articulate remarkable characteristics of conductivity, which relates to $\Pi ^{(\ell )}_{zz}(\omega ,q; T)$ as Eq. (19). For fixed *q* at room temperature ($T=300\,{\text{ K }}$), On the one hand, the real part of $\Pi ^{(\ell )}_{zz}(\omega ,q;T)$ closes to zero so that we can neglect such part. In the other hand, the imaginary part of the variable is considerable. Therefore, It is important to study the behavior of the frequency-dependent electrical conductivity tensor $\sigma _{\alpha \beta }(q=0)$ that depends on the imaginary part of the variable as presented in Fig. 3. From the Fig. 3a, we find that the first local minimum of $\Pi ^{(0)}_{zz}(\omega ,0;T)$ is above $-0.3\,{\text{ eV }}$. Hence, the value of the first local minimum increases when the chiral angle decrease for the quantization lines $\ell =0$. We observe that as $|\omega |$ increases, the poles decrease to zero. In the opposite, the imaginary part of $\Pi ^{(1)}_{zz}(\omega ,q;T)$ increases. In the Fig. 3b, the first local minimum of the imaginary part of $\Pi ^{(1)}_{zz}(\omega ,0;T)$ is above $0.5\, {\text{ eV }}$. For example, the zigzag (0,4) (blue lines), at the first local minimum $\omega =0.489\, {\text{ eV }}$, we have $\Pi ^{(1)}_{zz}(\omega ,0;T)\approx -0.018$. For the chiral (2,6) (green lines), at the first local minimum $\omega =0.413\,{\text{ eV }}$, we have $\Pi ^{(1)}_{zz}(\omega ,0;T)\approx -0.117$. Finally, the first local minimum $\omega =0.539\,{\text{ eV }}$, we have $\Pi ^{(1)}_{zz}(\omega ,0;T)\approx -0.097$ for the armchair (4,4) (red lines). According to the Fig. 3c the quantization lines $\ell =-1$, $\Pi ^{(-1)}_{zz}(\omega ,0;T)$ has the same curve characteristic as for the zigzag (0,4) (blue lines) and the armchair (4,4) (red lines) but it has a little change from $\Pi ^{(1)}_{zz}(\omega ,0;T)$ for the chiral (2,6) (green lines).

**Figure 4:**
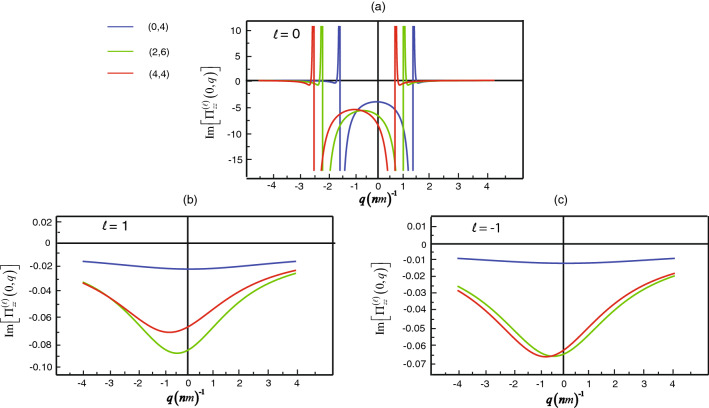
The simulation plots for the imaginary part of the current–current correlations $\Pi ^{(\ell )}_{zz}(0,q;T)$ relating to the wave number *q* in the zigzag (0,4) (blue lines), the chiral (2,6) (green lines), and the armchair (4,4) (red lines) with different orbital number where (**a**) $\ell =0$, (**b**) for $\ell =1$ and (**c**) for $\ell =-1$.

Then, we will describe the dc conductivity at room temperature via taking the limit $\omega =0$ for the tensor in which there is the ratio between the complex variable $\Pi ^{(\ell )}_{zz}(\omega ,q; T)$, and frequency. Although the frequency is close to zero, the conductivity is not undefined because the complex variable is close to zero as well. We calculate the imaginary part of $\Pi ^{(\ell )}_{zz}(\omega ,q; T)$ for the electron spin up parallel to the z-axis. Initially, we consider the quantization lines $\ell$ to be zero that show the behavior of the imaginary part of $\Pi ^{(\ell )}_{zz}(\omega ,q; T)$ as a function of *q*. In Fig. 4a, Such graphs are multiple maxima spikes, rapidly changing from positive to a negative value of $\Pi ^{(0)}_{zz}(0,q; T)$. The absolute of a rapidly changing point increase when the chiral angle increase. For example, the zigzag (0,4) (blue lines), the spikes is $q=\pm 1.55\,{\text{ nm }}^{-1}$. For the chiral (2,6) (green lines), the spikes is $q=1.114\,{\text{ nm }}^{-1}$ and $-2.30\,{\text{ nm }}^{-1}$. Finally, the spikes is $q=0.78\,{\text{ nm }}^{-1}$ and $-2.66\,{\text{ nm }}^{-1}$ for the armchair (4,4) (red lines). Also, they possess asymmetric properties in which negative and positive wave vectors corresponds with the shift of the Dirac point band structure according to Fig. 2a.

In the case of orbital motion, the imaginary part of the correlation function $\Pi ^{(\pm 1)}_{zz}(0,q;T)$ is significant since as Eq. (19), the conductivity tensor is as a result of unit imaginary multiply with the function that consists of a negative imaginary value so that the conductivity is composed of real value. Now we will consider the imaginary part of $\Pi ^{(\pm 1)}_{zz}(0,q;T)$. In Fig. 4b,c, $\Pi ^{(-1)}_{zz}(0,q;T)$ demonstrate curve characteristic that resemble $\Pi ^{(1)}_{zz}(0,q;T)$ for the chiral (2,6) (green lines) and the armchair (4,4) (red lines) but rather different from $\Pi ^{(1)}_{zz}(0,q;T)$ for the zigzag (0,4) (blue lines). For Fig. 4b,c, all lines vary different when the electron has an orbital characteristics of a spiral clockwise $(\ell =1 )$ and counterclockwise $(\ell =-1 )$. For instance, at the minimum point of the chiral (2,6) in Fig. 4c, there is $26.5 \%$ different from the minimum point of the same chiral index in Fig. 4b. The characteristics of the graphs imply that the electron which is the spin-up state consists of two different conductivity depending on the orbital motion. Even if the values of the conductivity are not equal in both orbital numbers, the tendencies of conductivity’s curves are similar. The orbital of a spiral counterclockwise $(\ell =-1 )$ has overall DC conductivity less than clockwise $(\ell =1 )$ for the electron in the spin-up state but it is usually opposite for the electron in the spin-down state. Consequently, if electrons moving through SWCNT become polarized, the backscattered electrons will be polarized in the opposite direction. The electron transmission in SWCNT, as a result, has the spin-polarized characteristic in carrier transport owing to the chirality of SWCNT.

**Figure 5:**
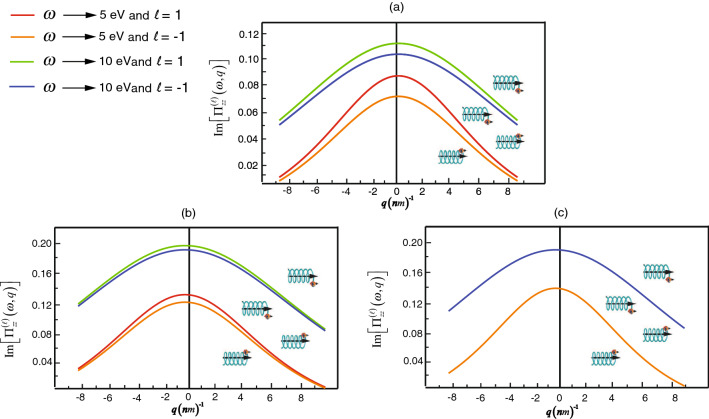
The simulation plots for the imaginary part of the current–current correlations $\Pi ^{(\ell )}_{zz}(\omega ,q;T)$ relating to the wave number *q* with specific frequencies and orbital numbers in (**a**) the zigzag (0,4), (**b**) the chiral (2,6) and (**c**) the armchair (4,4).

Now we will consider characteristics of the spin-polarized for each SWCNT in Fig. 5. From the simulational calculation, it implies that conductivity of the electron’s spin-up state which is based on the correlation split to two lines for different of orbital $(\ell =1 )$ and $(\ell =-1)$. For example, there is a maximum difference at $q=0\,{\text{ nm }}^{-1}$ for the zigzag (0,4) and at $q=-0.53\, {\text{ nm }}^{-1}$ for the chiral (2,6). The maximum difference for the zigzag (0,4) as Fig. 5a is about $0.015 (\omega =5 \, {\text{ eV }})$ and $0.0078 (\omega =10 \, {\text{ eV }})$ and the maximum difference, for the chiral (2,6) as Fig. 5b is 0.0069 $(\omega =5 \, {\text{ eV }})$ and $0.0028 (\omega =10 \, {\text{ eV }})$. Nevertheless, in the case of armchair (4,4), there is no conductivity’s spitting. Our theoretical results inform the chirality effects on SWCNT that if SOI caused by the distance between the interacting atoms projected on the tube’s axis exists (such as the Zigzag and the Chiral), there is the separation of the conductivity. The strength of SOI considerably depends on the distance $d^{||}_{\delta }$ in Eq. (6). On the contrary, in the case of the armchair in which the distance close to zero, the separation of the conductivity vanish.

**Figure 6:**
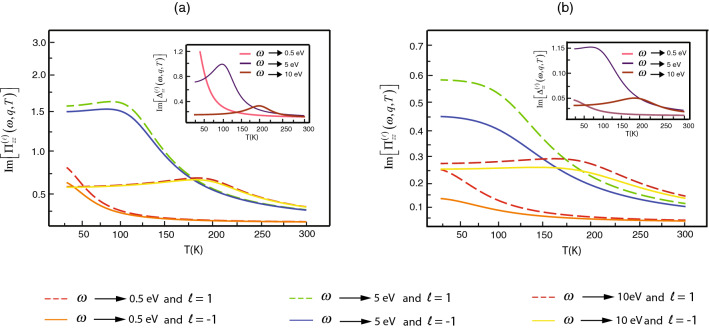
The simulation plots for the imaginary part of the current–current correlations $\Pi ^{(\ell )}_{zz}(0,q;T)$ relate to the temperature *T* with different orbital numbers where the wave number $q = 1\;{\text {nm}}^{-1}$ and inserted plots are the imaginary part difference of the correlations in (**a**) the zigzag and (**b**) the chiral.

The certain parameters from Eq. (28) that affect the phenomena are (1) a spiral clockwise $(\ell =1)$ or counterclockwise $(\ell =-1)$ of the electron motion, (2) the chiral index of SWCNT, and (3) temperature which is shown in Fig. 6. To explore the typical temperature dependence, we will consult Fig. 6a,b that are the representation orders of the magnitude difference from the imaginary part of $\Pi ^{(\pm 1)}_{zz}(\omega ,q;T)$ at temperature range 10–300 K. The figures demonstrate that the splitting of conductivity implied by the magnitude difference dwindles when the temperature increase. This result corresponds to the recent experiment of Rahman^39^ for the inversion asymmetric helical potential of DNA creating a spin-filtering effect, which polarizes carrier spins in the nanotube.

Such a tendency indicates that the phenomenon may disappear in the high-temperature case. From the figures, we observe the characteristics of inserted graphs which signify conductivity’s separation that graphs in the high-frequency case are nonmonotonic where the maximum conductivity’s spitting emerges at the specific critical temperature. We regard further for the high-frequency respect that if the electron’s frequency increase, the critical temperature increase. On the other hand, where the electron’s frequency is not high, the graph characteristic is rather monotonic decay in the zigzag but might be slightly nonmonotonic in the chiral. This effect is due to the convolution of current–current correlation defined as a thermal average. As a result, the critical temperature is shifted according to the responding frequency of conductivity. The theoretical results imply spin polarization estimated by the different imaginary part of the current–current correlation being two phases. In the first phase where the temperature is less than the critical temperature, increasing spin polarization corresponds with the increasing temperature. Nonetheless, in the later phase where the temperature is more than the critical temperature, the result is opposite that increasing temperature leads to decreasing spin polarization. The correlation function, therefore, is highest at the pole where thermal energy relates to responding frequency of conductivity in the case of nonmonotonic. However, in the case of the low frequency, the nonmonotonic characteristic disappear since the kinetic energy of the electron’s current is so low that the pole most occurs where the temperature close to zero. The theoretical characteristic of first phase insinuates physical meaning that the thermal energy causes the electrons to transfer from the valence band to the bottom of conduction bands which are different from the velocities $v^{(K)}_{L/R}$ in the different orbital. Consequently, in the first phase, increasing temperature induces the separation of conductivity. In the second phase, however, thermal energy boosts electrons to higher conduction bands in which their velocities are a little different. The separation of conductivity is somewhat not explicit since most electrons in each orbital have the close energy. Accordingly, in the later phase, increasing temperature decreases the separation of conductivity. The effects of SOI in our model is analogous to the Zeeman effect that the presence of the magnetic field makes a spectral line spilt into several components. If orbital and spin of the electron are the opposite directions, the produce magnetic potential is positive according to the nature of SOI^44^. Such potential cause the backscattering effect, hence overall electron’s movement impedes. On the contrary, where orbital and spin of the electron are the same direction, the induced magnetic potential is negative. The negative potential lead to reduced backscattering effect. By the different orbital with the same spin direction, the conductivity of SWCNT separate via the unequal backscattering effect.

## Conclusions

Our research investigates the characteristics of conductivity of carbon nanotubes of three types of single-wall carbon nanotubes by using the Green-Kubo method and thoroughly considering the tide-binding model. We derive the fascinating characteristics of the longitudinal current–current correlation function which leads to many peculiar attributes that the left-going $v^{(K)}_{L}$ and the right-going $v^{(K)}_{R}$ velocity consist of asymptotic in conduction and valence bands possessing a linear dispersion, see in Fig. 2. To study the conductivity attribute, we emphasize especially on the imaginary part of the current–current correlation function which indicates implicitly overall conductivity. We found out that there are different correlations between different orbitals. In addition, we discovered the temperature dependence in the different imaginary parts of the current–current correlation which is nonmonotonic at certain frequencies. We perceived that if responding frequencies of conductivity are high, the nonmonotonic attribute appears. We discerned that in the obvious nonmonotonic case, the difference between the imaginary part of the correlation exists to be two phases which are increasing function phase and decreasing function phase relating to the temperature. Such the difference is maximum at a certain critical temperature. However, in the low responding frequency case, the critical temperature is close to zero, then their nonmonotonic property likely vanishes. The results of the phenomena investigated from our study express different degrees of spin polarization in each chiral of single-walled carbon nanotube and significant effects on temperature-dependent charge transport according to carrier backscattering. This work raises the intriguing possibility of engineering charge transport in nanotubes via chiral-induced spin selectivity produced frp, each chiral index of SWCNT and is applied for fascinating optimization charge transport.

## Methods

### Tight-binding model and current operator for a single-walled carbon nanotube with chirality

Let us analyze SOI effects on the motion of electron spin up, defining the spin orientations “up” and “down” as parallel and antiparallel to the z-axis. We focus on the analytical calculation of energy band structures and the correlation function for each nanotube’s chiral index (n,m) as the armchair SWCNT, (4,4), and the zigzag SWCNT, (0,4) and the chiral SWCNT (2,6). The honeycomb lattice can be described in term of two triangular sublattices A and B. The unit vector of the triangular sublattice are $\overrightarrow {a} _{1}  = \frac{a}{2}(\sqrt 3 ,1)$ and $\overrightarrow {a}_{2}=\frac{a}{2}(\sqrt{3},-1)$ with $a=\sqrt{3}a_{cc}$ and $a_{cc}$ is the distance between two nearest carbon atoms. Any A atom is connected to its nearest-neighbors on B sites by the three vectors $\overrightarrow {\delta } _{i}$: $\vec{\delta }_{1}  = \frac{1}{3}\left( {\overrightarrow {a} _{1}  + \overrightarrow {a} _{2} } \right)$ and $\vec{\delta }_{2}  = \frac{1}{3}\left( { - 2\overrightarrow {a} _{1}  + \overrightarrow {a} _{2} } \right)$ and $\overrightarrow {\delta } _{3}  = \frac{1}{3}\left( {\overrightarrow {a} _{1}  - 2\overrightarrow {a} _{2} } \right)$. For discusing the electronic structure of the chiral SWCNT, we consider the tight-binding Hamiltonian with $N_c$ lattice sites $\overrightarrow {R}$ and nearest-neighbor lattice vector $\overrightarrow {\delta } _{i}$,

1 $$\begin{aligned} {\hat{H}}_{0}=\sum ^{N_{c}}_{ \overrightarrow {R}}\sum _{ \overrightarrow {\delta }_{i}}[t_{ \overrightarrow {R}, \overrightarrow {R}+ \overrightarrow {\delta }_{i}}{\hat{a}}^{\dagger }_{ \overrightarrow {R}}{\hat{b}}_{ \overrightarrow {R}, \overrightarrow {R}+ \overrightarrow {\delta }_{i}}+H.c.] \end{aligned}$$

when $t_{ \overrightarrow {R}, \overrightarrow {R}+ \overrightarrow {\delta }}$ is the hopping parameters between neighboring sites. We can completely determine the geometry of SWCNT by using a pair of integers (*n*, *m*), which denote the relative position of pair of atoms on graphene strip, $\overrightarrow {C}_{mn} = n \overrightarrow {a}_{1}+m \overrightarrow {a}_{2}$. Each SWCNT can be uniquely defined by its roll-up, $\overrightarrow {C}_{mn}$. This chiral vector $\overrightarrow {C}_{mn}$ defines the circumference of the tube axis. The roll-up vector connects two atoms on the graphite sheet which, once rlled up to form a nanotube, are mapped onto each other. The radius $\rho (n,m)$ and the chiral angle $\theta (n,m)$ of the tube are respectively given as

2 $$\begin{aligned} \rho (n,m)=\frac{a}{2\pi }\sqrt{n^2+nm+m^2}, \quad \theta (n,m)=arccos\left[ \frac{2n+m}{2\sqrt{n^2+nm+m^2}}\right] , \end{aligned}$$

The chiral angle $\theta (n,m)$ is in the range $0\le \theta (n,m) \le 30^\circ$, because of the hexagonal symmetry of the graphene lattice. This chiral angle also denotes the tilt angle of the hexagons concerning the direction of the nanotube axis. Zigzag tubes of the type $\theta (n,0)=30^\circ$ exhibit a zigzag pattern along the circumference. For metallic SWCNT, the armchair tube of the type $\theta (n,n)=0^\circ$ exhibits an armchair pattern along the circumference. Both zigzag and armchair nanotube is the achiral tube, in contrast with general chiral carbon nanotube, where $m \ne n \ne 0$.

Let us consider the dispersion relation tilted by a curvature effect of the nanotube. Three special results are discussed (1) the curvature of the surface of the SWCNT modifies the hopping integral between $\pi$ electrons from the flat graphene and (2) curvature induces $\sigma -\pi$ mixing. (3) Following the method used by Ref.^32–34^, the modification of the $2p_{z}$ atomic orbitals, presenting the spin–orbit coupling (SOI), is included. In this way, the tight-binding Hamiltonian of carbon nanotube with the Fourier components,

3 $$\begin{aligned} {\hat{a}}_{ \overrightarrow {R}}=\int \frac{d^{2} \overrightarrow {k}}{(2\pi )^2}e^{i \overrightarrow {k}\cdot  \overrightarrow {R}}{\hat{a}}_{ \overrightarrow {k}}\quad {\hat{b}}_{ \overrightarrow {R}}=\int \frac{d^{2} \overrightarrow {k}}{(2\pi )^2}e^{i \overrightarrow {k}\cdot  \overrightarrow {R}}{\hat{b}}_{ \overrightarrow {k}} \end{aligned}$$

can be written based on the Bloch function for the *A* and *B* sublattices as

4 $$\begin{aligned}  \hat{H}_{0}  = \int {\frac{{d^{2} \overrightarrow {k} }}{{(2\pi )^{2} }}} \left( {\begin{array}{*{20}l}    {\hat{a}_{{\overrightarrow {k} }}^{\dag } } \hfill & {\hat{b}_{{\overrightarrow {k} }}^{\dag } } \hfill  \\   \end{array} } \right)\left( {\begin{array}{*{20}l}    0 \hfill & {\hat{h}_{{AB}}^{{(0)}} } \hfill  \\    {\hat{h}_{{AB}}^{{\dag (0)}} } \hfill & 0 \hfill  \\   \end{array} } \right)\left( {\begin{array}{*{20}l}    {\hat{a}_{{\overrightarrow {k} }} } \hfill  \\    {\hat{b}_{{\overrightarrow {k} }} } \hfill  \\   \end{array} } \right)  \end{aligned}$$

where ${\hat{h}}^{(0)}_{AB}$ is the single particle Hamiltonian

5 $$\begin{aligned} {\hat{h}}^{(0)}_{AB}=\sum _{ \overrightarrow {\delta }_{i}}e^{i \overrightarrow {k}\cdot  \overrightarrow {\delta }_{i}} \left( \begin{array}{ll} t_{11}( \overrightarrow {\delta }_{i}) &{}\quad t_{12}( \overrightarrow {\delta }_{i})\\ t_{21}( \overrightarrow {\delta }_{i}) &{}\quad t_{22}( \overrightarrow {\delta }_{i}) \end{array} \right) \end{aligned}$$

and $t_{ij}( \overrightarrow {\delta }_{i})$ is the hopping parameters following Ref.^21^

6 $$ \begin{aligned}   t_{{ij}} ({\overrightarrow {\delta }}_{i} ) &  = \left( {V_{{pp}}^{\pi } \left( {1 - \frac{1}{2}\left( {\frac{{d_{\delta }^{ \bot } }}{\rho }} \right)^{2} } \right)} \right) - (V_{{pp}}^{\sigma }  - V_{{pp}}^{\pi } )\frac{{(d_{\delta }^{ \bot } )^{4} }}{{4a_{{cc}} \rho ^{3} }}\sigma _{0}  + \left( {V_{{pp}}^{\pi } \left( {\frac{{d_{\delta }^{ \bot } }}{\rho }} \right) - (V_{{pp}}^{\sigma }  - V_{{pp}}^{\pi } )\frac{{(d_{\delta }^{ \bot } )^{3} }}{{2\rho a_{{cc}}^{2} }}} \right)\sigma _{z}  \\     & \quad  + i\frac{{\kappa _{{SO}} (d_{\delta }^{ \bot } )^{2} }}{{\rho a_{{cc}}^{2} }}d_{\delta }^{\parallel } (V_{{pp}}^{\sigma }  - V_{{pp}}^{\pi } )\left( {1 - \frac{1}{8}\left( {\frac{{d_{\delta }^{ \bot } }}{\rho }} \right)^{2} } \right)\left( {cos\tilde{\theta }\hat{\sigma }_{x}  - sin\tilde{\theta }\hat{\sigma }_{y} } \right) \\  \end{aligned}  $$

where ${\hat{\sigma }}_{j};\;j=x, y,z$ are the component of the Pauli matrix and ${\hat{\sigma }}_{0}$ is the identity matrix, defining as

7 $$\begin{aligned} \sigma _{0}=\left( \begin{array}{ll} 1&{}\quad 0\\ 0&{}\quad 1 \end{array} \right) ,\, \sigma _{x}=\, \left( \begin{array}{ll} 0&{}\quad 1\\ 1&{}\quad 0 \end{array} \right) ,\, \sigma _{y}=\left( \begin{array}{ll} 0&{}\quad -i\\ i&{}\quad 0 \end{array} \right) ,\, \sigma _{z}=\left( \begin{array}{ll} 1&{}\quad 0\\ 0&{}\quad -1 \end{array} \right) . \end{aligned}$$

here $d^{\parallel }_{\delta }$ and $d^{\perp }_{\delta }$ are the components of the nearest-neighbor lattice vector $\overrightarrow {\delta }$ in the basis of direction perpendicular and parallel to the tube axis and ${\tilde{\theta }}=\frac{1}{2}(\theta _{i}+\theta _{j})$. The hopping between two neighboring $p_{z}$ orbitals at sites “i” and “j” as $V^{\sigma }_{pp}$ and $V^{\pi }_{pp}$ are the transfer integrals giving rise to $\sigma$ and $\pi$ orbitals in flat two dimensional graphence, respectively, and $\kappa _{SO}$ is a dimensionless parameter indicating the SOI strength.

Since we are interested in the current response, the vector potential $\overrightarrow {A}$ is introduced in the Hamiltonian Eq. (3) by means of the Peierls substitution $\overrightarrow {k}\rightarrow  {\overrightarrow {k}}+\frac{e}{c} \overrightarrow {A}$, which introduces the phase factor $exp(\frac{e}{hc}i \overrightarrow {\delta }_{i}\cdot  \overrightarrow {A})$ . We expand the Hamiltonian Eq. (3) to second order in the vector potential, one has

8 $$\begin{aligned} {\hat{h}}_{AB}={\hat{h}}^{(0)}_{AB}-\sum _{ \overrightarrow {\delta }_{i}}\frac{e}{c} \overrightarrow {A}\cdot  \overrightarrow {\delta }_{i}e^{i \overrightarrow {k}\cdot  \overrightarrow {\delta }_{i}} \left( \begin{array}{ll} t_{11}( \overrightarrow {\delta }_{i})&{}\quad t_{12}( \overrightarrow {\delta }_{i})\\ t_{21}(\overrightarrow {\delta }_{i})&{}\quad t_{22}( \overrightarrow {\delta }_{i}) \end{array} \right) +\frac{1}{2} \sum _{ \overrightarrow {\delta }_{i}}\left( \frac{e}{c}\right) ^2 \overrightarrow {A}\cdot  \overrightarrow {\delta }_{i}e^{i \overrightarrow {k}\cdot  \overrightarrow {\delta }_{i}} \left( \begin{array}{ll} t_{11}( \overrightarrow {\delta }_{i})&{}\quad t_{12}( \overrightarrow {\delta }_{i})\\ t_{21}( \overrightarrow {\delta }_{i})&{}\quad t_{22}( \overrightarrow {\delta }_{i}) \end{array} \right)  \overrightarrow {\delta }_{i}\cdot  \overrightarrow {A}. \end{aligned}$$

The total current density operator is obtained by differentiating Eq. (5) with respect to $\overrightarrow  {A}$ as

9 $$\begin{aligned} {\hat{j}}_{\alpha }=-e\partial _{\alpha }{\hat{h}}^{(0)}_{AB}={\hat{j}}^{(p)}_{\alpha }+{\hat{j}}^{(d)}_{\alpha } \end{aligned}$$

Here ${\hat{j}}^{(p)}_{\alpha }$ and ${\hat{j}}^{(d)}_{\alpha }$ are the paramagnetic and the diamagnetic current density operator, respectively

10 $$\begin{aligned} {\hat{j}}^{(p)}_{\alpha } =\sum _{\overrightarrow  {\delta }_{i}}\frac{1}{c}\delta ^{(\alpha )}_{i}e^{i \overrightarrow {k}\cdot  \overrightarrow {\delta }_{i}} \left( \begin{array}{ll} t_{11}( \overrightarrow {\delta }_{i})&{}\quad t_{12}( \overrightarrow {\delta }_{i})\\ t_{21}( \overrightarrow {\delta }_{i})&{}\quad t_{22}( \overrightarrow {\delta }_{i}) \end{array} \right) \end{aligned}$$

and

11 $$\begin{aligned} {\hat{j}}^{(d)}_{\alpha } =\frac{1}{2}\sum _{ \overrightarrow {\delta }_{i}}\frac{1}{c}\delta ^{(\alpha )}_{i}\delta ^{(\beta )}_{i}e^{i \overrightarrow {k}\cdot  \overrightarrow {\delta }_{i}} \left( \begin{array}{ll} t_{11}( \overrightarrow {\delta }_{i})&{}\quad t_{12}( \overrightarrow {\delta }_{i})\\ t_{21}( \overrightarrow {\delta }_{i})&{}\quad t_{22}( \overrightarrow {\delta }_{i}) \end{array} \right) A_{\beta }, \end{aligned}$$

where $\alpha$ and $\beta$ are dummy indices.

We can expand the Hamiltonian Eq. (5) and the current density operator Eqs. (10) and (11) around the Dirac point, $\overrightarrow  {K}$ and $\overrightarrow {K'}$, as we are interested in the physics taking place around $\mu$ the Femi energy. The choice of $\overrightarrow  {K}=\frac{4\pi }{3\sqrt{3}a_{cc}}(-1,0)$ and $\overrightarrow {K'}=\frac{4\pi }{3\sqrt{3}a_{cc}}(1,0)$ allows us to write the two independent Fermi point in a compact form as $\overrightarrow  {K}\tau$ with $\tau =+1$ for $-\overrightarrow  {K}$ and $\tau =-1$ for $\overrightarrow {K'}$. For the expansion of Eq. (5), we substitute $\overrightarrow {k}=\tau  \overrightarrow {K}+ \overrightarrow {q},  \overrightarrow {k}$, being small enough to justify the expansion $e^{i \overrightarrow {k}\cdot  \overrightarrow {\delta }_{i}}\approx e^{i\tau  \overrightarrow {K}\cdot  \overrightarrow {\delta }_{i}}(1+i \overrightarrow {\delta }_{i}\cdot  \overrightarrow {q})$. With the definitions

12 $$\begin{aligned} \Delta k^c_{\perp }=\frac{a_{cc}}{4{\rho }^2}\tau \left( 1+\frac{3}{8}\left( \frac{V^\sigma _{pp}-V^\pi _{pp}}{V^\pi _{pp}}\right) \right) cos3\theta ,\quad \Delta _{flip}=\frac{\kappa _{SO}}{4\rho }\left( \frac{V^\sigma _{pp}-V^\pi _{pp}}{V^\pi _{pp}}\right) , \end{aligned}$$

and

13 $$\begin{aligned} \Delta k^{so}_{\perp }=\frac{2\kappa _{SO}}{\rho }\left( 1+\frac{3}{8}\left( \frac{V^\sigma _{pp}-V^\pi _{pp}}{V^\pi _{pp}}\right) \right) ,\quad \Delta k^c_{\parallel }=-\frac{a_{cc}}{4{\rho }^2}\tau \left( 1+\frac{5}{8}\left( \frac{V^\sigma _{pp}-V^\pi _{pp}}{V^\pi _{pp}}\right) \right) sin3\theta , \end{aligned}$$

we can write ${\hat{h}}^{(0)}_{AB}$ as

14 $$\begin{aligned} {\hat{h}}^{(0)}_{AB}= \hslash v_{F}e^{-i\tau \theta }\left( \tau (q_{\perp }+\Delta k^c_{\perp })-i(q_{\parallel }+\tau \Delta k^c_{\parallel })\right) {\hat{\sigma }}_{0}- \hslash v_{F} e^{-i\tau \theta }\Delta k^{so}_{\perp }{\hat{\sigma }}_{y}-i \hslash v_{F}e^{-i\tau \theta }\Delta _{flip}\left( cos{\tilde{\theta }}{\hat{\sigma }}_{x}-cos{\tilde{\theta }}{\hat{\sigma }}_{z}\right) \end{aligned}$$

where $v_{F}=\frac{3{\pi }a_{cc}}{h}{\mid }V^{\pi }_{pp}|\approx 8.6 \times 10^{5}{\text{ m }}\;{{\text{ s }}^{-1}}$ is the group velocity at the Fermi point. The non-interacting single Hamiltonian Eq. (9) has the eigenvalue as

15 $$\begin{aligned} E_{\pm }( \overrightarrow {q}) =\pm \hslash v_{F}\left( (q_{\perp }+\Delta k^{c}_{\perp })^2+(q_{\parallel }+\tau \Delta k^{c}_{\parallel })^{2} +(\Delta k_{\perp }^{so})^{2}+\Delta _{flip}^{2} +2\sigma \left( (\Delta k_{\perp }^{so})^{2}+(q_{\parallel }+\tau \Delta k^{c}_{\parallel })^2\Delta _{flip}^{2} +(\Delta k_{\perp }^{so})^{2}(q_{\perp }+\Delta k^c_{\perp })^2\right) ^{\frac{1}{2}}\right) ^{\frac{1}{2}}. \end{aligned}$$

In addition, a boundary condition is imposed that yield the appropriate quantization of the vector $\overrightarrow {q}$. The SWCNT is obtained by rolling a graphene layer into a tube, in the angular direction its wave function always obey periodic boundary condition $\Psi ( \overrightarrow {C}_{nm})=exp(i \overrightarrow {q}\cdot  \overrightarrow {C}_{nm})\Psi (0)=e^{i2{\pi }\ell }\Psi (0)$ . This boundary condition leads to a quantization of the transverse component of the vector $\overrightarrow {q},\quad q_{\perp }=2\pi \ell /\mid  \overrightarrow {C}_{nm}\mid$ , with $\ell$ being an integer.

For the dispersion relation Eq. (18), we are interested in the asymmetric velocities obtained by differentiating Eq. (18) with respect to $q_{\parallel } \rightarrow q$ as

16 $$\begin{aligned} v^{(K)}_{L}=-\frac{1}{\hslash }\frac{\partial E( \overrightarrow {q})}{\partial q}\quad ( q < 0), v^{(K)}_{R}=\frac{1}{\hslash }\frac{\partial E( \overrightarrow {q})}{\partial q} \,( q > 0.) \end{aligned}$$

The asymmetric velocities can be approximated as

17 $$\begin{aligned} v^{(K)}_{L}=v_{F} - \frac{1}{2}\sigma v_{F}\Delta _{flip},\quad v^{(K)}_{R}=v_{F} +\frac{1}{2}\sigma v_{F}\Delta _{flip} \end{aligned}$$

where the velocity difference between the left- and right-going velocities in the linear band $\Delta v$ is given as

18 $$\begin{aligned} \Delta v= \sigma v_{F}\Delta _{flip} =\sigma \frac{3\kappa _{SO}{\pi }a_{cc}}{4\rho h}|V^{\pi }_{pp}|\left( \frac{V^\sigma _{pp}-V^\pi _{pp}}{V^\pi _{pp}}\right) . \end{aligned}$$

here $\sigma$ is a spin index ($+1$ for a spin up and $-1$ for a spin down), it implies that effect of SOI on charge transport depending on the spin electron in SWCNT.

### Electrical conductivity

Let us consider the current density operator for the paramagnetic and diamagnetic contribution Eq. (6). The frequency-dependent electrical conductivity tensor $\sigma _{\alpha \beta }(\omega )$ is calculated using the Kubo formula^45^

19 $$\begin{aligned} \sigma _{\alpha \beta }(\omega )=\frac{i}{\omega }\left( \Pi _{\alpha \beta }(\omega )+\frac{ne^2}{m}\delta _{\alpha \beta }\right) \end{aligned}$$

where $\Pi _{\alpha \beta }(\omega )$ is the retarded correlation function for current is given by

20 $$\begin{aligned} \Pi _{\alpha \beta }(\omega )=\int ^{\infty }_{-\infty }dt e^{i\omega t}\Pi ^R_{\alpha \beta }(t) \end{aligned}$$

and

21 $$\begin{aligned} \Pi ^R_{\alpha \beta }(t)=-\frac{i}{A}\Theta (t)Tr({\hat{\rho }}{\hat{j}}_{\alpha }(\overrightarrow  {q},t){\hat{j}}_{\beta }(\overrightarrow  {q},0)). \end{aligned}$$

here ${\hat{\rho }}=exp[-{\hat{H}}/k_{B}T]/Z$ is the density matrix of the canonical ensemble, $Z=Tr[exp[-{\hat{H}}/k_{B}T]]$ is the partition function at temperature T, and ${\hat{j}}_{\alpha }(t)$ are the current density operator as

22 $$\begin{aligned} {\hat{j}}_\alpha (t)=e^{\frac{i}{\hslash }{\hat{H}}t}{\hat{j}}_\alpha ( {q},0)e^{-\frac{i}{\hslash }{\hat{H}}t}. \end{aligned}$$

For the particle density of the lattice model

23 $$\begin{aligned} {\hat{n}}(\overrightarrow  {q})=\sum _{\overrightarrow  {k}}\left( {\hat{a}}^\dagger ( \overrightarrow {k}){\hat{a}}^\dagger ( \overrightarrow {k}+ \overrightarrow {q})+{\hat{b}}^\dagger ( \overrightarrow {k}){\hat{b}}^\dagger ( \overrightarrow {k}+ \overrightarrow {q})\right) \end{aligned}$$

the charge density ${\hat{\rho }}_{c}( \overrightarrow {q})=e \overrightarrow {n}( \overrightarrow {q})$ obeys the continuity equation $\dot{\hat{\rho }}_{c} (\vec{q}) - \vec{q} \cdot \widehat{{\overrightarrow {j} }}(\vec{q}) = 0$ for the paramagnetic current operator. We can thus consider this operator to be the paramagnetic current operator of the lattice model for general $\overrightarrow {q}$. It is usually easiest to calculate the retarded correlation function in $G(t, \overrightarrow {k})$ the Matsubara Green’s function formalism,

24 $$\begin{aligned} G(t, \overrightarrow {k})=-Tr\left( {\hat{\rho }}\left( \begin{array}{ll}{\hat{a}}( \overrightarrow {k},t)\\ {\hat{b}}( \overrightarrow {k},t)\end{array}\right) \left( \begin{array}{ll}{\hat{a}}^\dagger ( \overrightarrow {k},0)&{\hat{b}}^\dagger (\overrightarrow  {k},0)\end{array}\right) \right) \end{aligned}$$

Thus we can rewrite $\Pi _{\alpha \beta }(\omega )=Tr[{\hat{\rho }}{\hat{j}}_\alpha (\overrightarrow  {q},t){\hat{j}}_\beta (\overrightarrow  {q},0)]$ in terms of the matrix element of the current operator ${\hat{j}}_{\alpha }(t=0)$, and the Matsubara Green’s function, by using the representation for the retarded current–current correlation function can find in the form

25 $$\begin{aligned} \Pi ^{(\ell )}_{\alpha \beta }(\omega , \overrightarrow {q};T) = e^2\sum _{\omega _{n}} Tr\left( \partial _\alpha {\hat{h}}_{AB}^{(0)}( \overrightarrow {q})G(\omega _n, \overrightarrow {q}) \partial _\beta {\hat{h}}_{AB}^{(0)}(\overrightarrow  {q}) G(\omega _n-\omega, \overrightarrow  {q}) \right) . \end{aligned}$$

where the Fourier transformation of Matsubara Green’s function, $G(\omega _n, \overrightarrow {q})$, is given as

26 $$\begin{aligned} G(\omega _n, \overrightarrow {q})= \frac{(i \omega _n+\mu )\sigma _{0}+\sigma _{+}{\hat{h}}_{AB}^{(0)}+\sigma _{-}{\hat{h}}_{AB}^{(0)*}}{(i\omega _n+\mu )^2-E^2( \overrightarrow {q})}, \end{aligned}$$

and $\omega _n=(2n+1)\pi k_B T.$ Now we consider the longitudinal correction function $\Pi _{zz}(\omega ,\overrightarrow {q})$ for the lowest order of energy dissipation. Inserting Eq. (19) to the retarded current–current correlation function Eq. (28), It is possible to rewrite Eq. (28) in the form

27 $$\begin{aligned} \Pi ^{(\ell )}_{zz}(\omega ,\overrightarrow  {q};T) = \pi ^{(R)}(\omega , \overrightarrow {q};\sigma )\Theta (q) + \pi ^{(L)}(\omega ,\overrightarrow  {q};\sigma )\Theta (q) . \end{aligned}$$

here $\Theta (\overrightarrow  {q})$ is the Heaviside step function and $\pi ^{(R(L))}(\omega , \overrightarrow {q};\sigma )$ the left- and right-going correlation function is given as

28 $$\begin{aligned} \pi ^{(R(L))}(\omega , \overrightarrow {q};\sigma ) =e^2\sum _{\omega _{n}} Tr\left( \left( v_{R(L)}^{(K)}\right) ^2 G(\omega _n, \overrightarrow {q}) G(\omega _n-\omega , \overrightarrow {q}) \right) . \end{aligned}$$

This equation corresponds to the axial conductivity derived in Ref.^20^ by using the Boltzmann kinetic equation. In the opposite case, Eqs. (16) and (17) contain the effect of SOI giving a different result for a spin up and down and antisymmetric of the left- and right-going correlation function, which not appear in the axial conductivity derived in Ref.^20^. Because of antisymmetric velocity between the left- and right-going shows that asymmetric charge transport in the lattice structure of SWCNT as same as to calculate the local temperatures and heat currents we find the stationary state by solving a system of algebraic equations^46^.

## References

[1] Miura R (2014). Ultralow mode-volume photonic crystal nanobeam cavities for high efficiency coupling to individual carbon nanotube emitters. Nat. Commun..

[2] Laird EA (2015). Quantum transport in carbon nanotubes. Rev. Mod. Phys..

[3] Rosati R, Dolcini F, Rossi F (2015). Electron-phonon coupling in metallic carbon nanotubes: Dispersionless electron propagation despite dissipation. Phys. Rev. B.

[4] Li Z, Bai B, Dai Q (2016). Efficient photo-thermionic emission from carbon nanotube arrays. Carbon.

[5] Mori T, Yamauchi Y, Honda S, Maki H (2015). An electrically driven, ultrahigh-speed, on-chip light emitter based on carbon nanotubes. Nano Lett..

[6] Miyauchi Y (2014). Tunable electronic correlation effects in nanotube-light interactions. Phys. Rev. B.

[7] Fan Y, Singer SB, Bergstrom R, Regan BC (2009). Probing planck's law with incandescent light emission from asingle carbon nanotube. Phys. Rev. Lett..

[8] Miyauchi Y (2013). Photoluminescence studies on exciton photophysics in carbon nanotubes. J. Mater. Chem. C.

[9] Fujiwara M, Tsuya D, Maki H (2013). Electrically driven, narrow-linewidth blackbody emission from carbon nanotube microcavity devices. Phys. Rev. Lett..

[10] Barkelid M, Steele GA, Zwiller V (2012). Probing optical transitions in individual carbon nanotubes using polarized photocurrent spectroscopy. Nano Lett..

[11] Singer SB, Mecklenburg M, White ER, Regan BC (2011). Polarized light emission from individual incandescent carbon nanotube. Phys. Rev. B.

[12] Hsu H, Reichl LE (2007). Selection rule for the optical absorption of graphene nanoribbons. Phys. Rev. B.

[13] Chung HC, Lee M, Chang CP, Lin MF (2011). Exploration of edge-dependent optical selection rules for graphene nanoribbons. Opt. Express.

[14] Sasaki K-I, Kato K, Tokura Y, Oguri K, Sogawa T (2011). Theory of optical transitions in graphene nanoribbons. Phys. Rev. B.

[15] Hsien-Ching C, Cheng-Peng C, Chiun-Yan L, Ming-Fa L (2016). Electronic and optical properties of graphene nanoribbons in external fields. Phys. Chem. Phys.

[16] Fantini C (2009). Investigation of the light emission efficiency of single-wall carbon nanotubes wrapped with different surfactants. Chem. Phys. Lett..

[17] Thiti T, Watchara L, Tula J, Boonchui S (2017). Curvature effect on polarization of light emitted from chiral carbon nanotubes. Opt. Express.

[18] Charlier JC, Lambin P (1998). Electronic structure of carbon nanotubes with chiral symmetry. Phys. Rev. B.

[19] Maiti A, Svizhenko A, Anantram M (2002). Electronic transport through carbon nanotubes: Effects of structural deformation and tube chirality. Phys. Rev. Lett..

[20] Slepyan GY, Maksimenko SA, Lakhtakia A, Yevtushenko O, Gusakov AV (1999). Electrodynamics of carbon nanotubes: Dynamic conductivity, impedance boundary conditions, and surface wave propagation. Phys. Rev. B.

[21] Yevtushenko OM, Slepyan GY, Maksimenko SA, Lakhtakia A, Romanov DA (1997). Nonlinear electron transport effects in a chiral carbon nanotube. Phys. Rev. Lett..

[22] Ivchenko EL, Spivak B (2002). Chirality effects in carbon nanotubes. Phys. Rev. B.

[23] Liu C-C, Jiang H, Yao Y (2011). Low-energy effective hamiltonian involving spin–orbit coupling in silicene and two-dimensional germanium and tin. Phys. Rev. B.

[24] Zhuang HL, Singh AK, Hennig RG (2013). Computational discovery of single-layer iii–v materials. Phys. Rev. B.

[25] Zhao M (2015). Driving a gaas film to a large-gap topological insulator by tensile strain. Sci. Rep..

[26] Dabsamut K, Thienprasert J, Jungthawan S, Boonchun A (2019). Stacking stability of c2n bilayer nanosheet. Sci. Rep..

[27] Saito T, Nugraha ART, Hasdeo EH, Hung NT, Izumida W (2019). Electronic and optical properties of single wall carbon nanotubes. Top. Curr. Chem..

[28] Izumida W, Vikström A, Saito R (2012). Asymmetric velocities of dirac particles and vernier spectrum in metallic single-wall carbon nanotubes. Phys. Rev. B.

[29] Izumida W, Okuyama R, Yamakage A, Saito R (2016). Angular momentum and topology in semiconducting single-wall carbon nanotubes. Phys. Rev. B.

[30] Konstantin P, Nikolaevich MP, Rashid NG (2014). Spin–orbit effects in carbon nanotubes–Analytical results. Phys. Rev. B.

[31] Gandil M, Matsuda K, Lounis B, Tamarat P (2019). Spectroscopic signatures of spin–orbit coupling and free excitons in individual suspended carbon nanotubes. Phys. Rev. B.

[32] Tsuneya A, Spivak B (2002). Spin–orbit interaction in carbon nanotubes. J. Phys. Soc. Jpn..

[33] Tsuneya A, Spivak B (2005). Theory of electronic states and transport in carbon nanotubes. J. Phys. Soc. Jpn..

[34] Valle M, Marganska M, Grifoni M (2011). Signatures of spin–orbit interaction in transport properties of finite carbon nanotubes in a parallel magnetic field. Phys. Rev. B.

[35] Kuemmeth F, Ilani S, Ralph DC, McEuen PL (2013). Coupling of spin and orbital motion of electrons in carbon nanotubes. Nature.

[36] Steele GA (2013). Large spin–orbit coupling in carbon nanotubes. Nature.

[37] Jae SJ, Hyun WL (2009). Curvature-enhanced spin–orbit coupling in a carbon nanotube. Phys. Rev. B.

[38] Asadpour SH (2017). Goos-hänchen shifts due to spin–orbit coupling in the carbon nanotube quantum dot nanostructures. Appl. Opt..

[39] Rahman MW (2020). Carrier transport engineering in carbon nanotubes by chirality induced spin polarization. ACS Nano.

[40] Jakubka F (2013). Mapping charge transport by electroluminescence in chirality-selected carbon nanotube networks. ACS Nano.

[41] Chang C-W, Okawa D, Majumdar A, Zettl A (2006). Solid-state thermal rectifier. Science (New York, N.Y.).

[42] Azadeh S (2014). Thermal rectification of a single-wall carbon nanotube: A molecular dynamics study. Solid State Commun..

[43] Tsuneya A (2000). Spin–orbit interaction in carbon nanotubes. Phys. Soc. Jpn..

[44] Winkler R (2003). Spin–Orbit Coupling Effects in Two-Dimensional Electron and Hole Systems.

[45] Gerald MD (2000). Many-Particle Physics.

[46] Simón MA, Martínez-Garaot S, Pons M, Muga JG (2019). Asymmetric heat transport in ion crystals. Phys. Rev. E.

